# The Antivivisectionist

**Published:** 1908-06

**Authors:** 


### EDITORIALS

The Business Office of the JOURNAL-RECORD is 1 1-2 to 5 1-2 South Broad St.
The Editorial Office is 1014-15 Century Bldg.
Address all Business Communications to Journal-Record of Medicine, 1 1-2 to 5 1-2 South Broad Street.
Make remittances payable to THE JOURNAL-RECORD OF MEDICINE.
On matters pertaining to the Editorial and Original communications, address Edgar G. Ballenger, M. D., Atlanta, Ga.
Reprints of original articles will be furnished at cost price. Requests for the same should always be made in THE MANUSCRIPT.
We will present, postpaid, on request, to each contributor of an original article, twenty (20) marked copies of THE JOURNAL-RECORD OF MEDICINE containing such article.

The Medical Journal Company has purchased Dr. George Brown's interest in the Journal-Record of Medicine. With this disposal of his stock, Dr. Brown resigns as business manager of the Journal-Record, and extends to each subscriber and advertiser his thanks for favors shown and hopes for a continuation of their valued support of the Journal-Record of Medicine.

### THE ANTIVIVISECTIONIST.

There are a few consistent opponents of vivisection, the best illustration of whom is to be found in that large mass of the people of India who do their best to avoid killing even a poisonous insect. Yet these, in spite of all precautions, destroy daily the lives of millions of minute beings. In the West there are great numbers of apparently normal persons who are not nearly so considerate of life in general as are these East Indians, and who yet raise their voices in fierce vituperation against the scientific vivisector who deals out less pain in a year than most hunters in a day, and indeed than a large proportion of stock dealers in a minute. We doctors are libelled as heartless wretches by ignorant or prejudiced upholders of the sanctity of the guinea pig in contradistinction to the cheapness of the human. We are supposed to care nothing for the sufferings of any creature besides man. We know that to the medical mind vivisection is a painful necessity and not out of accord with the methods whereby nature or the God of nature enlarges the gradual evolution of life. All good has come along a road which might be called evil, and was certainly imperfect. When we understand better, perhaps we will find that evil is nothing but one side of good. In any case a study of nature's processes makes it perfectly evident that to raise a huge outcry against a properly regulated system of vivisection having in view the annihilation of disease, pain, and even death in men and animals alike is to exhibit to the world a mind deficient in judgment and insight. The antivivisector, not the scientific vivisector, it is who deserves to be placarded as cruel and heartless as well as weak. But the missionary spirit of the opponent of vivisection is worthy of cultivation if turned into a useful channel. And the wonder is, if his love for the animal world is as acute as he would have us believe, that our cities continue to be infernos of cruelty. A walk through their streets sickens and disgusts. If the physiologist behaved to his necessary sacrifices on the holy altar of science as some dealers and owners of stock behave out of mere lack of sympathy and laziness to their helpless victims he would be worthy of all the worst that has ever been said of him. We daily pass crate upon crate of chickens dying for a drop of water and trampling upon one another in the stifling air of a burning pavement, and horses and mules whose every step is an agony being mercilessly urged by the whips of drivers merrily whistling while their beasts would fain lie down and die. Is not there something of the savage in us still, that such things should be permitted? And is not there a place for the medical profession in the ranks of the society for the prevention of cruelty to animals? Should not every doctor as a matter of course, belong to that society? S.

